# How to recognize and manage psychosomatic pain in the pediatric emergency department

**DOI:** 10.1186/s13052-021-01029-0

**Published:** 2021-03-25

**Authors:** Giorgio Cozzi, Annunziata Lucarelli, Fabio Borrometi, Ilaria Corsini, Eva Passone, Sara Pusceddu, Giuliana Morabito, Egidio Barbi, Franca Benini

**Affiliations:** 1 Pediatric Emergency Department, Institute for Maternal and Child Health IRCCS Burlo Garofolo, Via dell’Istria 65/1, 34137 Trieste, Italy; 2 Department of Paediatrics and Emergency, Giovanni XXIII Children’s Hospital, University of Bari, Bari, Italy; 3 Pediatric Pain Service and Palliative Care, Department of Oncology, Pausilipon Hospital, AORN Santobono Pausilipon, Naples, Italy; 4 Pediatric Emergency Unit, Department of Medical and Surgical Sciences (DIMEC), Sant’Orsola-Malpighi Hospital, University of Bologna, Bologna, Italy; 5 Pediatric Clinic, Department of Clinical and Experimental Sciences, DAME, ASUFC S. Maria Della Misericordia, University of Udine, Udine, Italy; 6 Pediatric Department, Ospedale S. Maria della Scaletta AUSL, Imola, Italy; 7 Pediatric and Neonatology Division, Azienda Ospedaliera Santa Maria degli Angeli, Pordenone, Italy; 8 Department of Medical, Surgical and Health Sciences, University of Trieste, Trieste, Italy; 9 Paediatric Palliative Care – Pain Service Department of Women’s and Children’s Health, University of Padua, Padua, Italy

**Keywords:** Adolescence, Pain, Pediatric emergency department, Somatic symptom disorder

## Abstract

**Aim:**

Children and adolescents affected by somatization and somatic symptom disorder commonly refer to emergency services. Due to the absence of specific guidelines for the emergency setting and to a possible lack of knowledge, these patients are at risk of being unrecognized and mismanaged. This study aims at proposing a clinical practice to approach and manage these patients and their families in the emergency setting.

**Methods:**

This manuscript derived from the work of a research group of italian pediatric emergency physicians and anesthesiologists, with an expertise in pain management, members of the PIPER group. The research group reviewed the literature about psychosomatic pain and somatic symptom disorder and developed a clinical practice specific for the pediatric emergency setting.

**Results:**

The manuscript provides information about the main clinical features shared by patients with psychosomatic pain and about current diagnostic criteria and appropriate management in the emergency setting. Furthermore, it highlights the possible pitfalls in which the emergency physician may run into dealing with these patients.

**Conclusion:**

This clinical practice should be seen as a starting point toward a better understanding of patients with psychosomatic pain and a standardization of care in the pediatric emergency setting.

## Background

The presence of physical symptoms inconsistent with a clear physical disease is usually defined as somatization [1, 2]. When physical symptoms are long-lasting and negatively impact on patients’ daily activities a mental health disorder may be present. Somatic symptom disorder (SSD) is a mental health disorder in which affected subjects report long-lasting physical symptoms and these symptoms are associated with considerable distress and disruption of daily functioning [3]. Patients with somatization and SSD commonly refers to the emergency services because of their symptoms. They represent a significant part of daily practice for physicians working in the pediatric or general emergency department (ED) and at primary care services. EDs are busy settings in which these patients, who commonly complain of long-lasting symptoms, may receive low priority at triage, and it may take a long time from their arrival to the medical evaluation. Furthermore, due to a possible lack of awareness and training of physicians, these patients risk not being recognized, misdiagnosed and mismanaged. In this contest, inappropriate investigations can reinforce the vicious circle of worry about an unknown disease, increase anxiety and, ultimately contribute to doctor shopping.

ED physicians should be aware of how challenging these patients are and should care for these patients and their families appropriately. Nevertheless, no clinical practices or guidelines addressing these patients at the ED are available.

PIPER (Pain in Paediatric Emergency Room) group is an Italian study research network that collects members from 52 Italian emergency departments. Participants are pediatricians, emergency physicians, and anesthesiologists who work together to share knowledge and research in pain recognition, assessment and management, from an emergency setting point of view [4, 5]. Every year, the members meet in a plenary session and discuss which aspects of the management of patients complaining of pain symptoms are more challenging and deserving more knowledge and attention in order to improve and standardize patient’s care in the Italian pediatric emergency setting. When a specific aspect is identified a working group is formed and works throughout the year, to develop a document that could be shared with the entire network. In consideration of the growing population of children and adolescents who arrives at the ED with mental health problems, the Piper Group set up a specific working group to develop a clinical practice for the care of children and adolescents with psychosomatic pain and SSD in the emergency department.

## Methods

The PIPER study group used the following model to develop this clinical practice. At first, during a plenary meeting of the PIPER group in Rome, September 2018, the issue of the recognition, assessment and appropriate management of psychosomatic pain and SSD at the ED was presented and discussed. At that time, the main features of patients with psychosomatic pain and SSD were indicated. The high prevalence of these conditions in the pediatric population, the frequent association with psychiatric comorbidities, were the common use of primary care, and emergency services of these patients were highlighted. Finally, the absence of guidelines for their management at the ED setting and the lack of standardization of care among institutions was emphasized and discussed. Therefore, it was considered necessary to develop a clinical practice. Coming from eight different Institutions, a working group of prominent pediatric and anesthesiologist researchers and leaders in pain management and functional and psychiatric pain syndromes was elected.

At first, an electronic medical search of PubMed, EMBASE and the Cochrane Library from January 2000 to June 2019, limited to English language studies, was carried out. The search syntax on PubMed was: children OR child OR adolescence OR adolescents AND psychosomatic OR somatoform OR somatic symptom disorder OR somatization OR functional somatic. We searched references of pertinent articles identified by our search strategy for additional relevant papers. We selected publications with an emphasis on the past 5 years, but we did not exclude older influential publications commonly referenced.

The clinical practice was structured as follows: - introduction with definitions, classification, and aims; − recognition and diagnosis; − recommended management in the ED; − possible pitfalls. The team members divided the tasks, providing the first draft of each section of the clinical practice and each part of the latter was discussed within the working group, allowing the creation of a first complete draft. Disagreements were discussed and solved with an open discussion of the practice. Furthermore, the clinical practice was independently revised by two child psychiatrists, national experts in SSD management. Their comments were discussed within the group used to implement the clinical practice. Then, the revised clinical practice was presented at the plenary meeting of the PIPER group in Rome, September 2019. After a thorough discussion and incorporating the suggestions from the audience, a final version of the clinical practice was resubmitted to all PIPER members for their final approval.

## Results

### Definitions and classification

Somatization is defined as the presence of a physical symptom inconsistent with a clear physical illness [1, 2]. Somatization is a common and, most of the times, benign event in childhood and adolescence [6]. However, when symptoms have a considerable negative impact on patients’ feelings and behaviours, somatization becomes a disorder. Pain is the most commonly reported symptom by patients with somatization or SSD [7, 8].

The Diagnostic and Statistical Manual of Mental Disorders fifth edition (DSM-5) defines the somatic symptom disorder (SSD) as a condition in which the patient’s subjective reporting of physical symptoms is associated with distress, disruption of daily functioning and disproportionate thoughts, feelings and behaviors related to such symptoms [3]. Table 1 shows the diagnostic criteria for somatic symptom and related disorders, according to the DSM-5. In children and adolescents with a marked limitation in daily activities lasting for at least 1 month, an SSD diagnosis can be made.

**Table 1:** Diagnostic and Statistical Manual of Mental Disorders 5th Edition diagnostic criteria for somatic symptom and related disorders

Somatic Symptom Disorders and Related Disorders	
Somatic Symptom Disorder:	
- One or more somatic symptoms	
- Excessive thoughts, feelings, or behaviors related to the somatic symptoms or other associated symptoms such as excessing thoughts regarding the seriousness of symptoms, anxiety about the symptoms, or excess time and energy devoted toward the symptoms.	
- The patient is persistently symptomatic and the somatic symptoms may change over time (typical duration of 6 months)	
- Specifiers: with predominant pain, persistent, mild, moderate, severe	
Illness Anxiety Disorder:	
- Preoccupation with having or acquiring illness	
- Somatic symptoms are either mild or not illness:	
- If a medical condition is present or there is a high risk of a medical condition, the preoccupation is excessive and disproportionate to the risk of illness	
- High level of anxiety about health	
- Performs excessive health-related behaviors or maladaptive avoidance	
- Preoccupation with illness lasting at least 6 months, although the specific illness that is feared may change over that time	
- Specifiers: care-seeking type, care-avoidant type	
Functional Neurologic Symptom Disorder (Conversion Disorder):	
- At least one symptom of altered voluntary motor or sensory function	
- Clinical findings are incompatible with patient clinical presentation	
- Specifiers:	
- with weakness/paralysis	
- with abnormal movement	
- with swallowing symptoms	
- with speech symptom	
- with attacks/seizures	
- with anesthesia/sensory loss	
- with special sensory symptom	
- with mix symptom	
- acute episode (< 6 months), persistent (> 6 months)	
- with psychological stressor, without psychological stressor	
Psychological Factors affecting General Medical Condition:	
- Presence of medical condition	
- Psychological or behavioral factors adversely affect the medical condition by potentially (1) interfering with treatment, (2) increasing health risk, (3) influencing underlying pathophysiology, and/or (4) close temporal association between these factors and exacerbation of illness	
- Specifiers: mild, moderate, severe, extreme	
Factitious Disorder:	
- Falsification of physical or psychological signs or symptoms associated with identified deception	
- Presents self to others as ill	
- Deceptive behavior can be present without identified external gains	
- Specifiers: single episode, recurrent episode, imposed on self or imposed on other	
**Shared features:**	
- Not better explained by another mental disorder or physical health condition	
- Symptoms cause significant impairment and/or distress	

### Reasons for a clinical practice

- Psychosomatic pain is a common occurrence in childhood and adolescence. Epidemiological studies show that 15–20% of children and adolescents refer to primary care due to somatization [9]. One study conducted at a tertiary level pediatric emergency department showed that 8.6% of children who complained of pain met the diagnostic criteria for SSD [10].
- Children and adolescents with psychosomatic pain and SSD show a worse quality of life, spend more days at home, miss more days of school, use the health care system more frequently when compared to healthy peers and to patients affected by organic diseases [6].
- Children and adolescents with psychosomatic pain and SSD, complain physical symptoms, thus they refer more frequently to primary care, emergency services and medical wards, than mental health services [6]. EDs may be the only place where these families seek medical assistance. Therefore, pediatricians and emergency physicians should be trained to identify, address, and manage these patients.
- An appropriate approach to these patients could reduce the inappropriate use of emergency services and related costs [11].
- To our knowledge, there are no clinical guidelines focused on the approach and management of patients with psychosomatic pain and SSD at the ED.

### Recognition and diagnosis

The diagnosis of somatization and SSD should be a positive diagnosis and not an exclusion one. ED physicians should be fully aware that this diagnosis should also be made in an ED setting. Validated and internationally recognized diagnostic criteria are available and should be used (Table 1).

Despite the absence of pathognomonic markers of these conditions, international evidence, based on epidemiological data and experts’ knowledge, shows that patients with somatization and SSD share some specific features which should be considered clues to the diagnosis [6–10, 12–19]:

- Adolescence age
- Female sex
- Presence of an already diagnosed chronic disease
- Presence of mild intellectual disability
- Previous psychiatric diagnosis, mainly anxiety disorder or depression
- History of violence, abuse or life adversities during childhood
- History of high family or social expectations on the subject
- History of high conflictual level inside the family
- Familiarity with psychiatric disorders
- History of chronic school absenteeism or bullying and victimization

The emergency physician should recognize the specific features of psychosomatic pain:

- Psychosomatic pain may be present in any part of the body, but is more commonly reported as headache or abdominal pain.
- It could be present simultaneously in multiple parts of the body or change localization with time.
- Usually, it starts as a recurrent pain and then it presents every day, and this continuous presence leads to a progressive limitation of the subject’s normal daily activities.
- Common analgesic drugs are ineffective, including opioids and adjuvants such as gabapentin and neuroleptics.
- It lasts for months, sometimes years, without a useful therapy.
- Frequently, it is associated with marked fatigue.

Patients with SSD present long-lasting physical symptoms, causing distress and considerable limitation of their everyday activities. The key to diagnosis is that these patients show a disproportionate functional impairment caused by their symptoms. They are unable to frequent school, pursue hobbies, practice sports. Usually, they have an impaired social life with peers and spend most of the time at home. They may develop a real disability made of incongruous medicalization or inappropriate use of medical aids such as wheelchair or crutches.

Functional impairment in these patients can negatively impact on the entire family, with parents spending much time dealing with their children’s symptoms.

Commonly, patients with somatization and SSD have a medical history remarkable for many already performed diagnostic tests and specialistic evaluations, sometimes in different facilities and cities. They may present a notable medical dossier at the visit, collecting the medical report of all their evaluations and tests. These features, as well as, a history of chronic school absenteeism should be considered as highly suggestive of SSD.

Therefore the emergency physician should actively ask about school attendance [14, 15]. A history of bullying or victimization should be taken in two account, because it is frequently present in patients with SSD.

A substantial percentage of patients with SSD have psychiatric comorbidity, primarily anxiety disorder or depression. Therefore, features suggestive of these conditions should be investigated [20].

The presence of an already diagnosed chronic disease should not be considered as an exclusion factor for the diagnosis [8]. On the contrary, according to the most recent diagnostic criteria, SSD diagnosis does not take into account the presence or absence of any organic disease. Moreover, the presence of a documented chronic illness should be thought of as a risk factor for SSD development [21].

Usually, the physical examination of these patients is unremarkable.

### Differential diagnosis

When symptoms last for a long time, without any red flag of an organic disease, with repeated diagnostic work-up already performed and with an unremarkable physical examination, the diagnosis of a previously unrecognized organic disease is highly unlikely. Nevertheless, the clinical history of every patient should be carefully assessed and every “red flag” for the presence of a superimposed organic disease should be taken into consideration. SSD’s primary differential diagnoses are factitious disorder and factitious disorder imposed by the caregiver [6, 7, 15, 22, 23].

### Recommended management in the ED

When unrecognized and untreated, SSD could be extraordinarily disabling and might lead to a progressive loss of life and social opportunities for intellectual growth. It may result in a poor adulthood outcome, leading to a permanent functional disability [24, 25]. Therefore, the ED setting can represent a unique window of opportunity to identify and support these patients.

At first, the ED physician should learn to actively ask patients and families the appropriate questions to highlight the amnestic features suggestive of somatization and SSD, especially in recognizing the disproportion between reported symptoms, physical examination, and significant functional limitation in daily activities caused by symptoms, accompanied by chronic school absenteeism and social withdrawal.

In case of highly suggestive history and clinical features, diagnostic tests to exclude organic diseases should be limited as much as possible, giving value to the already made diagnostic work-up results. These patients are commonly exposed to an incongruous medicalization and doctor shopping supported by families and even by physicians themselves [26]. Remarkably, the emergency physician should have the strength to oppose to an inappropriate request for further investigations and suggest additional diagnostic tests only when substantially required.

Emergency physicians should learn to communicate the diagnosis positively, according to the DSM-5 criteria. Patients and families can be reassured by their word, feeling relieved on the anxiety of suffering from un unknown disease. Reassurance may be enough in mild cases.

On the other hand, doctors should also consider that patient and families may not easily accept such a diagnosis. Therefore, they should dedicate time for explanations, and in some cases have a private conversation with the parents. When available, an evaluation with a child psychiatrist or psychologist could be helpful.

If an acceptance of the diagnosis seems unlikely, a referral to a dedicated service or a hospital admission should be suggested.

In any case, the diagnostic suspect should be mentioned in the ED medical record to facilitate the future physician’s approach and leave an overt trace of the physician’s conclusions. A diagnosis supported by a written explanation of its essential elements should be written on the discharge report.

SSD diagnosis requires a child psychiatrist confirm, but it could be hypothesized and expressed in an ED setting in front of a highly suggestive clinical picture. SSD patients require a multidisciplinary treatment that goes beyond the ED setting. Nevertheless, the emergency physician should know which are the cornerstones of treatment.

Psychological support is fundamental for these patients and families, and the emergency physician should suggest a psychological evaluation or refer them to a dedicated service [27, 28]. Psychotherapy aims to divert attention from symptoms, regain functioning and social life, look for possible trigger factors, and learn the coping strategies necessary to deal with this condition.

In general, as more time passes from the onset of symptoms to the diagnosis of SSD, the more severe the case and the higher the impairment level experienced by the patient. In these cases, a hospital admission will be required to activate multidisciplinary support with pediatricians, child psychiatrists, psychologists, nurses and physiotherapists to clarify the diagnosis and to start a funtional “rehabilitation” [27–31].

In the case of discharge, the ED physician should share the clinical opinion and diagnosis with the patient’s general practitioner or community pediatrician. General practitioners and community pediatricians will play a pivotal role with explanations, follow-up of patients and their families, and could guide the need for further specialistic evaluations. General practitioners and community pediatricians could also involve all the professional figures that work with children, such as psychologists, child psychiatrists, school teachers, sports trainers, occupational therapists and child’s life specialists, to coordinate these patients’ care.

Pharmacological treatment is not indicated for these patients, except for psychiatric comorbidities. Therefore, pharmacological therapies should be prescribed only after a careful psychiatric evaluation. Psychosomatic pain usually does not respond to common analgesics and neither to major opioids or adjuvants. Therefore, their use is not recommended [31, 32].

Furthermore, digital therapeutics and distraction technologies can be prescribed to help alleviate the symptoms that the patient may be experiencing.

### Possible pitfalls

- Missing to investigate the impact of symptoms on the patient’s daily activities, in patients with long-lasting physical symptoms.
  Remember to actively ask for school attendance, sport activities and social life with peers.
- Limit the evaluation to exclude an ongoing organic disease, not providing a positive diagnosis of SSD.
  The presence of an organic disease is not relevant for the diagnosis, and a negative diagnosis: “nothing is wrong in this child” is inappropriate and should be avoided.
- Prescribe analgesics. Pharmacological therapies and, in particular, analgesics are not useful for these patients and should be avoided.
- Not adequately communicating the diagnosis to patients and families, and not reporting it in the ED medical record, preferring a descriptive or vague description of symptoms. A presumptive diagnosis of SSD can be made in the ED setting, according to the criteria of the DSM-5.
- Lacking of awareness that a patient with SSD has a severe condition, a high risk of poor outcomes when not appropriately recognized and managed.
  Proper diagnosis in the ED setting could lay the foundation for the healing process. Conversely, a missed diagnosis could prompt families to search for a not understood or unknown disease, contributing to the persistence and amplification of symptoms and inappropriate medicalization.

## Discussion

Usually, emergency physicians and pediatricians working at the ED receive extensive education to recognize and treat medical conditions which can cause an immediate threat to the patients’ life. Fortunately, these cases are relatively rare, and most of the pediatric ED visits are related to non-urgent problems or minor urgencies.

Epidemiological studies have shown an ever-growing population of children and adolescents who come to ED for mental health problems [33, 34]. Most of the available evidence investigates how to approach children and adolescents with an immediate life risk, such as subjects with suicide attempts or ideation, self-harm, or agitation crisis and externalizing symptoms [35, 36].

A substantial percentage of patients with mental health problems complain of physical symptoms and for this reason the doctors should be trained to recognize those patients who hide a mental health problem behind a physical symptom. These patients are commonly affected by SSD. Usually, they are quiet and do not draw attention, with a high risk of being unrecognized. They do not run an immediate life risk, expect in the presence of psychiatric comorbidity, but they can face substantial morbidity and poor outcomes in adolescence and adulthood.

According to recent scientific publications, clinical practices can improve the clinical outcome of patients [36–38]. Despite the studies on SSD patients, who deal with their recognition and management inside and outside the hospital, there are no specific guidelines available to approach them at the ED [7, 13, 31, 37]. We believe this clinical practice can help appropriately recognize and appropriately manage these patients, outlining the necessary steps needed in ED.

Recently, a clinical pathway for SSD was developed by a working group of American psychiatrists [31]. It is a very useful document focused on managing admitted patients, but it does not refer specifically to the ED setting.

This clinical practice could lay the ground for the development of projects aimed to a change of organization of pediatric emergency settings to better deal with patients affected by mental health problems and specifically SSD. We believe that many steps should be made to improve the care of these patients in pediatric emergency settings. Patients with SSD frequently received low priority codes at ED triage, so a modification of the triage system could lead to a better estimation of the urgency of their visits. The knowledge of the ED health care staff about how to intercept and deal with SSD patients and their families should be strengthened and specific formation should be implemented. Dedicated spaces in the departments should be obtained to better receive these patients. The implementation of the availability in the ED of professional figures dedicated to the care of SSD patients such as psychologists and child psychiatrists could lead to a great improvement of the care of patients.

We are well aware that the recognition and management of patients affected by SSD at the ED is only a first step. These patients need to be cured properly inside and outside the hospital. In Italy, community services and territorial structures frequently have not enough resources to guarantee proper assistance to these patients and the availability of community child psychiatrists may not easily accessible. At the moment of the ED discharge, having the possibility to offer a scheduled control visit with a community professional could be helpful and improve short- and long-term assistance. In this sense, specific care pathways should be developed.

This work has some limitations. Our working group was composed of pediatricians and anesthesiologists experts in pain management. It did not include providers of other disciplines such as child psychiatrists or psychologists. Nevertheless, the clinical practice was evaluated by two child psychiatrists who have suggested some useful clues to approach these patients and it could lay the ground for an interdisciplinary implementation. This clinical practice provides general indications that should be adapted to different local settings and available resources. Further research studies are needed to investigate the practical utility of this tool in daily practice in the ED setting. This clinical practice should be seen as a starting point toward a better understanding of these patients and a standardization of care in the ED setting.

## Data Availability

Not applicable.

## Abbreviations

ED: Emergency department
SSD: Somatic symptom disorder
